# Petroleum pollution changes microbial diversity and network complexity of soil profile in an oil refinery

**DOI:** 10.3389/fmicb.2023.1193189

**Published:** 2023-05-23

**Authors:** Jugui Zhuang, Ruihuan Zhang, Yufei Zeng, Tianjiao Dai, Zhencheng Ye, Qun Gao, Yunfeng Yang, Xue Guo, Guanghe Li, Jizhong Zhou

**Affiliations:** ^1^State Key Joint Laboratory of Environment Simulation and Pollution Control, School of Environment, Tsinghua University, Beijing, China; ^2^School of Water Resources and Environment, China University of Geosciences, Beijing, China; ^3^State Key Laboratory of Urban and Regional Ecology, Research Center for Eco-Environmental Sciences, Chinese Academy of Sciences, Beijing, China; ^4^Institute for Environmental Genomics, University of Oklahoma, Norman, OK, United States; ^5^Department of Microbiology and Plant Biology, University of Oklahoma, Norman, OK, United States; ^6^Earth and Environmental Sciences Division, Lawrence Berkeley National Laboratory, Berkeley, CA, United States

**Keywords:** petroleum pollution, refinery, soil, microbial diversity, co-occurrence network

## Abstract

**Introduction:**

Petroleum pollution resulting from spills and leakages in oil refinery areas has been a significant environmental concern for decades. Despite this, the effects of petroleum pollutants on soil microbial communities and their potential for pollutant biodegradation still required further investigation.

**Methods:**

In this study, we collected 75 soil samples from 0 to 5 m depths of 15 soil profiles in an abandoned refinery to analyze the effect of petroleum pollution on soil microbial diversity, community structure, and network co-occurrence patterns.

**Results:**

Our results suggested soil microbial a-diversity decreased under high C10–C40 levels, coupled with significant changes in the community structure of soil profiles. However, soil microbial network complexity increased with petroleum pollution levels, suggesting more complex microbial potential interactions. A module specific for methane and methyl oxidation was also found under high C10–C40 levels of the soil profile, indicating stronger methanotrophic and methylotrophic metabolic activities at the heavily polluted soil profile.

**Discussion:**

The increased network complexity observed may be due to more metabolic pathways and processes, as well as increased microbial interactions during these processes. These findings highlight the importance of considering both microbial diversity and network complexity in assessing the effects of petroleum pollution on soil ecosystems.

## 1. Introduction

Petroleum pollutants from oil refinery areas have caused extensive contamination of soil and groundwater (Varjani, 2017; Khan et al., 2018). Previous studies report that ~6 million tons of oil seepage each year around the world (Kvenvolden and Cooper, 2001). More seriously, refined products contain more complicated components with a greater range of physicochemical properties than natural crude oil (Logeshwaran et al., 2018), including paraffin, cycloparaffins, and aromatics. Such refined products make pollution conditions in refinery areas even more complex and toxic. Furthermore, since the soil is a special porous medium with porosity and compressibility, most of the petroleum pollutants exhibit migration and diffusion processes in soil, resulting in shifts in their temporal and spatial distribution. Therefore, the toxicity caused by these petroleum pollutants can spread through the soil profile (Khan et al., 2018).

Soil microorganisms mediate biogeochemical cycles of carbon, nitrogen, phosphorus, sulfur, and various metals, and play critical roles in the degradation of petroleum pollution (Truskewycz et al., 2019). Many previous studies had investigated the effects of petroleum pollutants on soil microbial communities in refinery areas. Jiao et al. (2016) investigated bacterial community structure and co-occurrence network of soil samples in five oil refineries and illustrated the contribution of spatial distance to community variation and the non-random co-occurrence and ecological function-driven modular patterns of the network. Negative effects of TPH and heavy metals pollution were observed on bacterial community diversity in refinery areas (Dasgupta et al., 2018). Meanwhile, the microbial potential for pollutant biodegradation was identified in the soils of refinery areas (Arvanitis et al., 2008). Amini et al. (2017) isolated 18 phenanthrene- and pyrene-degrading bacteria from refinery soils and found three of them showed dioxygenase activity and produced biosurfactants. Microbial *alkB* and *bssA* genes for aerobic and anaerobic alkane degradation were detected in oily sludge from a refinery wastewater lagoon (Sarkar et al., 2016). However, most of these microbial studies only focused on the soil layer where spot pollution of petroleum occurs and ignored the soil layers where petroleum pollution migrates and diffuses. Therefore, little is known about the responses of soil microbial communities across the soil profile of petroleum pollution.

In this study, we collected 75 soil samples from 15 soil profiles in an abandoned refinery to assess the pollution level of different oil tank areas and its effect on soil microbial diversity, community structure, and co-occurrence patterns by using chemical analysis, high-throughput sequencing, and molecular ecological networks. Our study sheds light on the shifts in soil microbial diversity, community structure, network complexity, and the formation of network modules with specific ecological functions under high petroleum hydrocarbon pollution levels.

## 2. Materials and methods

### 2.1. Sample collection and measurement of soil parameters

In June 2021, we collected soil samples from an abandoned oil refinery in Henan province, China. A total of 15 boreholes were drilled at four different areas, namely, the heavy oil tank area, crude oil tank area, intermediate tank area (marked as site C1, site C2 and site C4 below), and finished oil tank area (marked as site C3 below). At each site of the three sites C1, C2, and C4, four boreholes (namely, D0–D3) were collected, but only three boreholes (namely, D1–D3) were collected at site C3 for the restriction of site conditions. In each borehole, five soil samples were collected from different depths of the soil profile ranging from 0.5 m to 5 m beneath the soil surface. A total of 75 soil samples were collected (Supplementary Table S1), and each sample was split into two parts for chemical analysis and DNA extraction, respectively. A range of soil parameters were analyzed, including general physicochemical characteristics, heavy metals, total petroleum hydrocarbon (TPH), BTEX, and PAHs, by using the related Chinese national standards.

### 2.2. DNA extraction

Soil total DNAs were extracted from 5 g of each soil sample by using CTAB/SDS method (Zhou et al., 1996). DNA concentration and purity were estimated by 1% agarose gel. In our experiment, high-quality soil total DNAs were obtained from 71 samples of the total 75 samples. These DNAs were stored at −80°C until sequencing analysis.

### 2.3. Amplicon sequencing and raw data analyses

The V4 hypervariable regions of 16S rRNA genes were amplified with the primer pair 515F (5′-GTGCCAGCMGCCGCGGTAA-3′) and 806R (5′ GGACTACHVGGGTWTCTAAT-3′). PCR products from different samples were purified and pooled at equal molality to be sequenced in the same run of the Illumina NovaSeq platform (Illumina, San Diego, CA, USA). In total, 250 bp paired-end reads were generated. Raw sequences were assigned to their corresponding samples according to the barcodes. After trimming primer and barcode sequences, paired-end reads were joined together. We performed quality control and dereplication of sequences by using usearch11 (Edgar, 2010). The high-quality sequences were processed to generate zero-radius operational taxonomic units (zOTUs) by UNOISE3 (Edgar, 2016). The representative 16S rRNA gene sequences were taxonomically annotated according to the Silva 16S rRNA gene database with a 50% confidence estimate. All sequences in each sample were randomly resampled to 37,800 sequences.

### 2.4. Statistical analyses

The richness, chao1, Simpson, and Shannon index were calculated to evaluate the α-diversities of soil bacterial communities using usearch11. Pearson's correlation, linear mixed-effect models were performed using R4.1.0 to explore the correlation between microbial α-diversity and environmental variables including pollutants. Non-parametric multivariate analysis of variance (Adonis) was used to test the differences in soil bacterial community structures, and principal coordinates analysis (PCoA) exhibited bacterial community structures of each sample based on the Bray–Curtis distance. Mantel test and redundancy analysis were also employed using R4.1.0 and related R packages to determine the linkage between environmental variables.

### 2.5. Network analyses

We constructed molecular ecological networks based on Random Matrix Theory (RMT) to reveal the possible co-existence patterns among soil microbes for sites C1, C2, and C4, respectively, by using the Molecular Ecological Network Analysis Pipeline (MENAP) (http://ieg4.rccc.ou.edu/MENA/) as previously described [Zhou et al., 2010; Deng et al., 2012]. Since obviously fewer samples were collected for site C3 than the other sites, no network for site C3 was constructed and compared with the other sites. For the network at each site, zOTUs detected in at least half of all samples were used for network construction, and Spearman correlations between each pair of zOTU were calculated to generate the correlation matrix. Network topological parameters were calculated to characterize the networks, including average degree (avgK), average clustering coefficient (avgCC), average path distance (GD), and harmonic geodesic distance (HD). Correlation between network modules and pollutants was also established by module-eigengene analyses. Finally, networks were visualized with Gephi0.9.7.

## 3. Results and discussion

### 3.1. Contaminants distribution and physicochemical properties of soil profiles

With all 15 sampling soil profiles, a total of 75 soil samples across different depths were analyzed from the abandoned oil refinery. Among the contaminants detected heavy metals such as Pb, Cd, Ni, As, and V, as well as long-chain petroleum hydrocarbons (C10–C40), showed a 100% detection rate (Table 1). Meanwhile, short-chain petroleum hydrocarbons (C6–C9) had a 72% detection rate, which is lower than C10–C40. Compared to C6–C9, C10–C40 consists of more complicated and resistant components and some of C10–C40 can be converted to short-chain C6–C9 products. Compounds of BTEX showed a detection rate ranging from 49.3% for benzene to 70.7% for m- and p-xylene. In the case of PAHs, only naphthalene showed a relatively high detection rate of 40% (Table 1). Compared to the Chinese National Standard, the refinery was mainly contaminated by organic contaminants, especially long-chain petroleum hydrocarbons. Specifically, 40% of samples had contaminations of C10–C40 exceeding risk screening values for soil contamination of the first category of development land, and 20% of samples had contaminations of C10–C40 exceeding risk intervention values of the first category of development land (Table 1). In addition, there are 17.3%, 22.7%, and 13.3% samples with contaminations of benzene, ethylbenzene, and naphthalene exceeding risk screening values for soil contamination of the first category of development land, respectively.

**Table 1:** Detection and exceed ratio of pollutants in the oil refinery areas.

		**Detection rate/%**	**Exceed ratio (risk screening value) /%**	**Exceed ratio (intervention value) /%**
Heavy metals	Pb	100	0	0
	Cd	100	0	0
	Ni	100	0	0
	As	100	0	0
	V	100	0	0
Petroleum hydrocarbons	C10–C40	100	**40**	**20**
	C6–C9	72	-	-
BTEX	Benzene	49.3	**17.3**	1.3
	Toluene	52	0	0
	Ethylbenzene	64	**22.7**	0
	m-&p- xylene	70.7	1.3	0
	o-xylene	52	0	0
PAHs	Naphthalene	40	**13.3**	0

The relatively high exceed ratio is highlighted by bold type.

**Figure 1:**
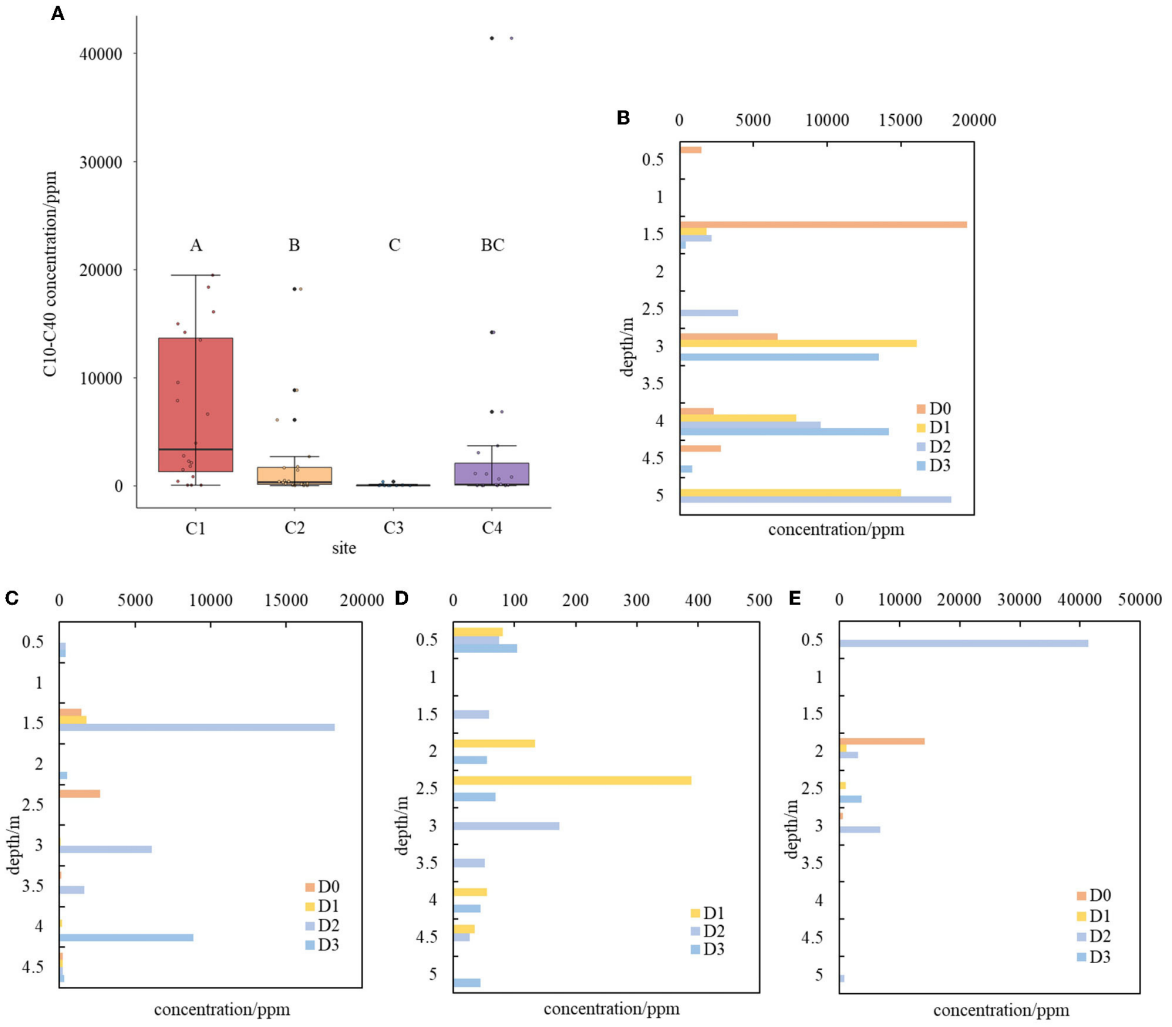
Distribution of C10–C40 contamination at different sites and along their soil profile **(A–E)**. **(A)** Distribution of C10–C40 contamination at different sites. Significances are denoted by letters. **(B–E)** Distribution of C10–C40 contamination along soil profile at site C1 **(B)**, site C2 **(C)**, site C3 **(D)**, and site C4 **(E)**.

The concentrations of C10–C40 in soils varied remarkably among the four sampling sites. The highest concentration of 41,400 mg/kg was found at site C4, followed by sites C1 and C2 with concentrations of 19,500 mg/kg and 18,200 mg/kg, respectively (Figure 1). In contrast, site C3 had the lowest C10–C40 concentration of 389 mg/kg (Figure 1). The pollution level at site C1 was significantly higher than those of all other three sites. Among the other three sites, only site C2 had a significantly higher pollution level than site C3, while no other significant differences were found. Furthermore, the concentrations of C10–C40 varied remarkably along the depths of soil profiles in the four sampling sites. At site C4, heavy C10–C40 pollution was mainly found in the shallow soils, while at site C2, it occurred at both 1.5 m and 4 m beneath the soil surface. At site C1, the heaviest pollution conditions were detected along soil depths ranging from 1.5 m to 5 m beneath the soil surface, indicating one or several heavy initial leakages of contaminants and their strong migration downward from the leakage point to deeper soil layers. These results indicated that long-chain petroleum hydrocarbon C10–C40 was the dominating pollutant and occurred in the soil profiles of the abandoned refinery.

Various geochemical characteristics were analyzed to determine their relevancy with organic contaminants (Supplementary Table S2). It was found that soil depth and soil organic carbon were positively correlated with the concentration of different kinds of organic contaminants, while CEC and the content of F, Al, and Fe were negatively correlated with some kinds of organic contaminants (*P* < 0.05, Pearson's correlation). Moreover, C10–C40 concentration was positively correlated with total nitrogen and $\text{N}{\text{O}}_{3}{}^{-}$-N, and negatively correlated with total phosphorus (*P* < 0.05, Pearson's correlation).

### 3.2. Soil microbial α-diversity variation

**Figure 2:**
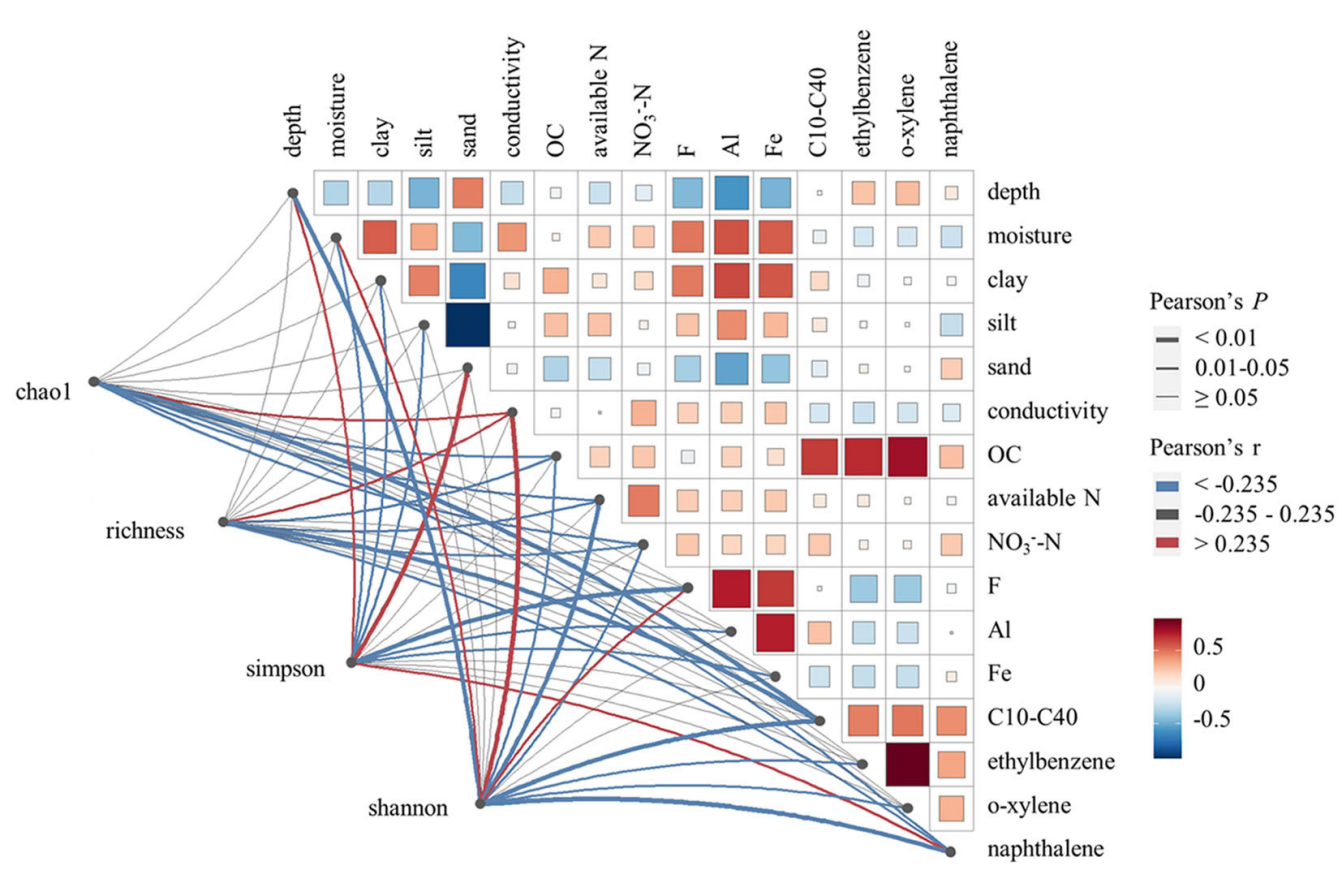
Pearson's correlation analysis between soil microbial α-diversity indexes and soil environmental variables. Only environmental variables showing significant correlations with soil microbial α-diversity are displayed in the figure.

The soil microbial α-diversity varied among samples, as indicated by diversity estimator indices such as richness, Chao1, Simpson, and Shannon. Pearson's correlation analysis (Figure 2) indicated that microbial richness and Chao1 diversity were positively correlated with soil conductivity, but negatively correlated with soil organic carbon, available nitrogen, $\text{N}{\text{O}}_{3}{}^{-}$-N, and contaminants such as C10–C40 and naphthalene (*P* < 0.05). Microbial Shannon diversity was positively correlated with soil moisture, conductivity, and F, but negatively correlated with soil depth, soil organic carbon, available nitrogen, $\text{N}{\text{O}}_{3}{}^{-}$-N, and contaminants such as C10–C40, ethylbenzene, o-xylene, and naphthalene (*P* < 0.05). In contrast, microbial Simpson diversity showed negative correlations with soil moisture, soil clay content, soil silt content, F, Al, and Fe, but positive correlations with soil depth, soil sand content, and the level of naphthalene (*P* < 0.05). Therefore, the same variation trend was observed among microbial richness, Chao1 diversity, and Shannon diversity, while Simpson diversity varied in a reverse trend. Hence, conductivity, soil organic carbon, available nitrogen, $\text{N}{\text{O}}_{3}{}^{-}$-N, and contaminants such as C10–C40 and naphthalene were identified as key factors affecting microbial α-diversity, with each showing a significant correlation with three or four α-diversity indices. However, we did not find any significant correlations between C6–C9 and any microbial α-diversity indices.

Soil depth has been proven to exert influence on various kinds of physicochemical characteristics, thus affecting soil microbial communities (Naylor et al., 2022). In our study, soil depth is found to be negatively correlated with soil moisture, conductivity, soil clay content, soil silt content, and inorganic elements including F, Al, and Fe, while positively correlated with soil sand content and some organic contaminants including ethylbenzene and o-xylene (*P* < 0.05). Therefore, soil depth may be a key environmental variable that affects soil microbial α-diversity both directly and indirectly. To test the variation of microbial diversity along soil profile, linear mixed-effect models were applied to further analyze the interaction effect of key environmental variables including soil depth and C10–C40 level on microbial α-diversity variation (Table 2). Richness was negatively correlated with both C10–C40 level and soil depth (*P* < 0.05). Furthermore, Chao1 diversity showed a negative correlation with the C10–C40 level (*P* < 0.05), and Shannon diversity was negatively correlated with soil depth (*P* < 0.05). Conversely, the Simpson diversity was positively correlated with soil depth. The reverse variation trend between the Simpson diversity and the other three α-diversity indexes was consistent with the results of Pearson's correlation analysis. For all microbial α-diversity indexes, no significant interaction effect of C10–C40 level and soil depth was observed. These results indicated that high pollution levels would lead to the decline of soil microbial α-diversity. The same result was also observed in similar studies (Camacho-Montealegre et al., 2021; Liu et al., 2022). For example, Liu et al. found that soil bacterial α-diversity was significantly lower under short-term petroleum pollution than under control conditions (Liu et al., 2022). Camacho-Montealegre *et al*. found that soil bacterial and fungal α-diversity decreased with increasing crude oil concentrations, while the relative abundance of microbes from different taxonomic groups showed different change patterns in response to external stress (Camacho-Montealegre et al., 2021). These changes could be due to varying resistance to the toxic effects of crude oil and changes in microbial predation activities.

**Table 2:** The effects of C10–C40 pollutant levels and soil depth on microbial α-diversity by linear mixed-effects models.

	**C10–C40**		**Depth**		**C10–C40** ^*^depth	
	**Coefficient**	***p*** **-value**	**Coefficient**	***p*** **-value**	**Coefficient**	***p*** **-value**
Chao1	−373.983	**0.049** ^*^	−226.525	0.102	−128.925	0.352
Richness	−279.042	**0.039** ^*^	−212.083	**0.046** ^*^	−68.083	0.521
Simpson	0.017	0.181	0.021	**0.004** ^**^	0.011	0.106
Shannon	−0.289	0.069	−0.312	**0.004** ^**^	−0.123	0.247

The linear mixed-effects models were performed in the following settings: α-diversity index ~ C10-C40 ^*^depth +(1|site). Significances are highlighted by bold type and denoted by asterisks: ^***^P < 0.001, ^**^0.001 ≤ *P* < 0.01, ^*^0.01 ≤ *P* < 0.05.

### 3.3. Pattern of soil microbial community structures

**Figure 3:**
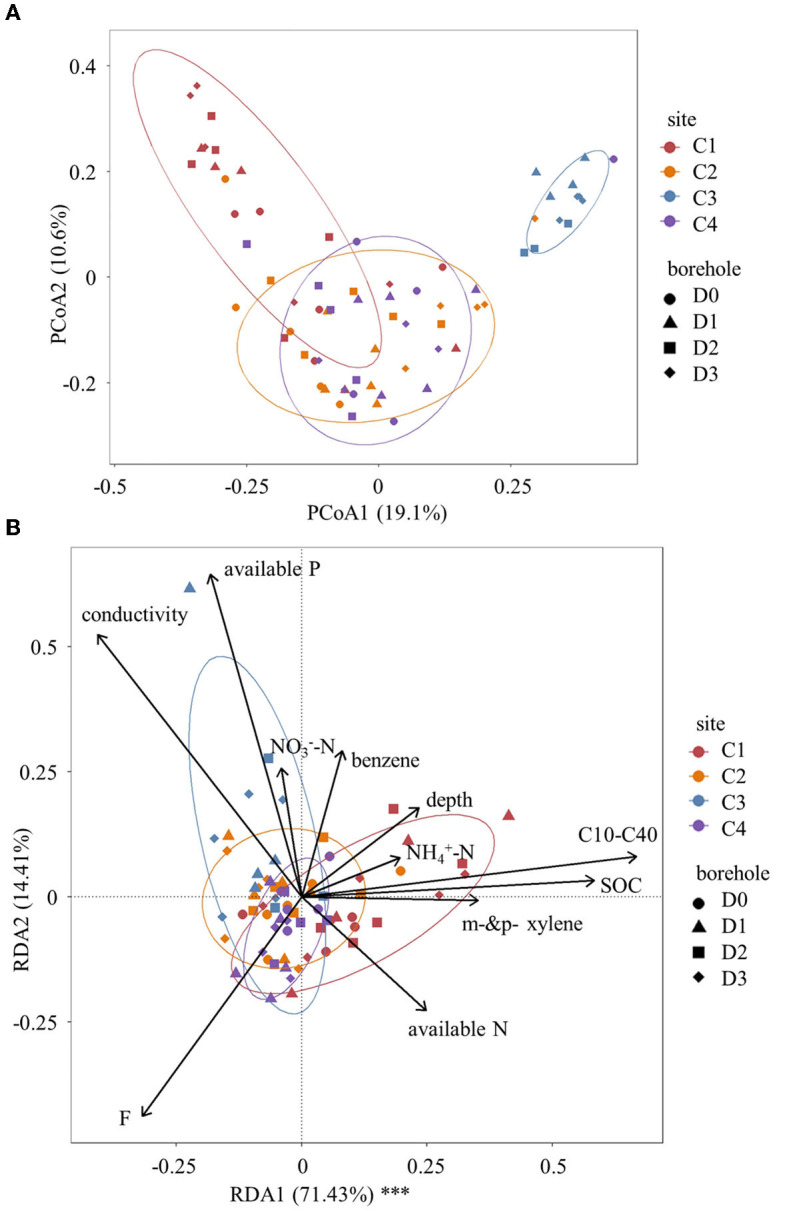
Shifts of soil community structure. **(A)** Principal coordinates analysis (PCoA) of soil microbial community structures based on Bray–Curtis distance. **(B)** Redundancy analysis (RDA) of soil microbial community structures. Significances are denoted by asterisks: ***P < 0.001, **0.001 ≤ *P* < 0.01, *0.01 ≤ *P* < 0.05.

Principal coordinates analysis (PCoA) plot revealed distinct differences in soil microbial community structures based on Bray–Curtis distance among the four sites (Figure 3A). Particularly for site C1 and site C3, they exhibited significantly different distributions from sites C2 and C4. Furthermore, the Adonis test was also used to test the interaction effect of site, soil depth, and C10–C40 level on microbial community structures (Table 3). The results indicated that microbial community structures based on Bray–Curtis distance were significantly affected by soil depth, C10–C40 level, and site (*P* < 0.05). However, no significant interaction effect of C10–C40 level and depth was observed at a P < 0.05 level. This result indicated that the influences of C10–C40 level on microbial community structures were independent of soil depth and did not significantly shift along soil depth. Moreover, pairwise Adonis (Supplementary Table S3) showed significant differences in microbial community structures based on Bray-Curtis distance among all sites (*P* < 0.01). These results were consistent with one previous study that soil microbial community structures were significantly shifted by sampling site and pollution level (Camacho-Montealegre et al., 2021).

**Table 3:** The effects of C10–C40 pollutant levels, soil depth, and sampling site on microbial community structure by non-parametric multivariate analysis of variance (Adonis).

	***R*-square**	***p*-value**
C10–C40	0.048	**0.001** ^***^
Depth	0.028	**0.004** ^**^
Site	0.188	**0.001** ^***^
C10–C40: depth	0.017	0.074

The non-parametric multivariate analysis of variance was performed by using the Adonis function in the following settings: Bray–Curtis dissimilarity ~ C10–C40 ^*^depth +(1|site). Significances are highlighted by bold type and denoted by asterisks.: ^***^P < 0.001, ^**^0.001 ≤ *P* < 0.01.

**Figure 4:**
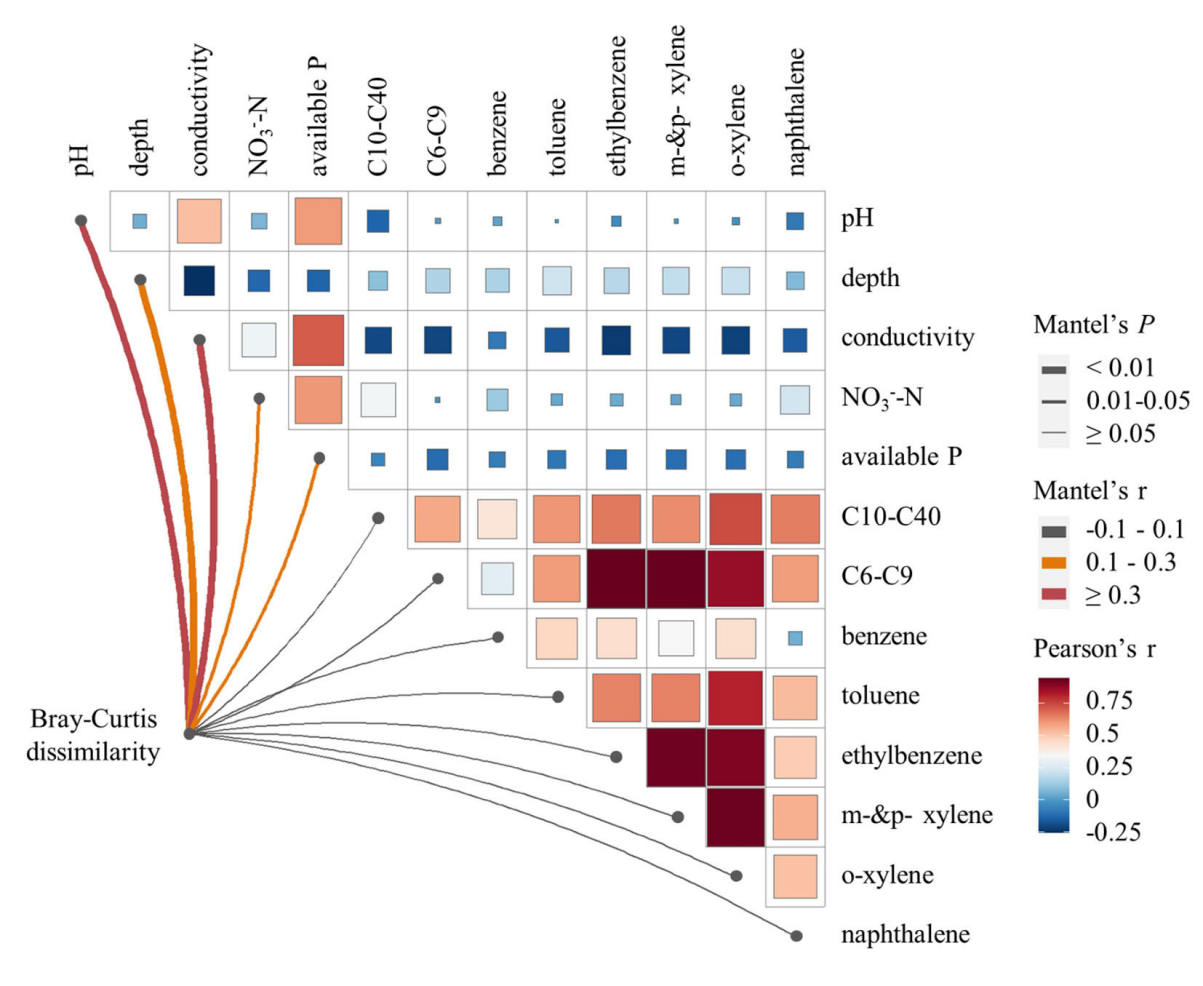
Mantel test of soil microbial community structures based on Bray–Curtis distance.

To determine the extent to which changes in soil microbial community could be explained by constrained environmental variables, we conducted a redundancy analysis (Figure 3B). The results demonstrated that environmental variables were highly explanatory of soil microbial community dissimilarity. Specifically, the RDA1 axis significantly explained 71.43% of the variance (*P* < 0.001), whereas the RDA2 axis explained 14.41% of the variance. The vector size of the environmental variables indicated that conductivity, F, available phosphorus, soil organic carbon, and C10–C40 level played crucial roles in explaining the changes in the soil microbial community. Based on the positions of the samples projected to the environmental variable vector, available phosphorus, and C10–C40 level emerged as the primary factors among these constrained environmental variables. Furthermore, the Mantel test (Figure 4) indicated that soil pH, depth, conductivity, NO${}_{3}^{--}$N, and available phosphorus were all found positively correlated with microbial communities based on Bray–Curtis distance (*P* < 0.05).

### 3.4. Soil molecular ecological network

**Figure 5:**
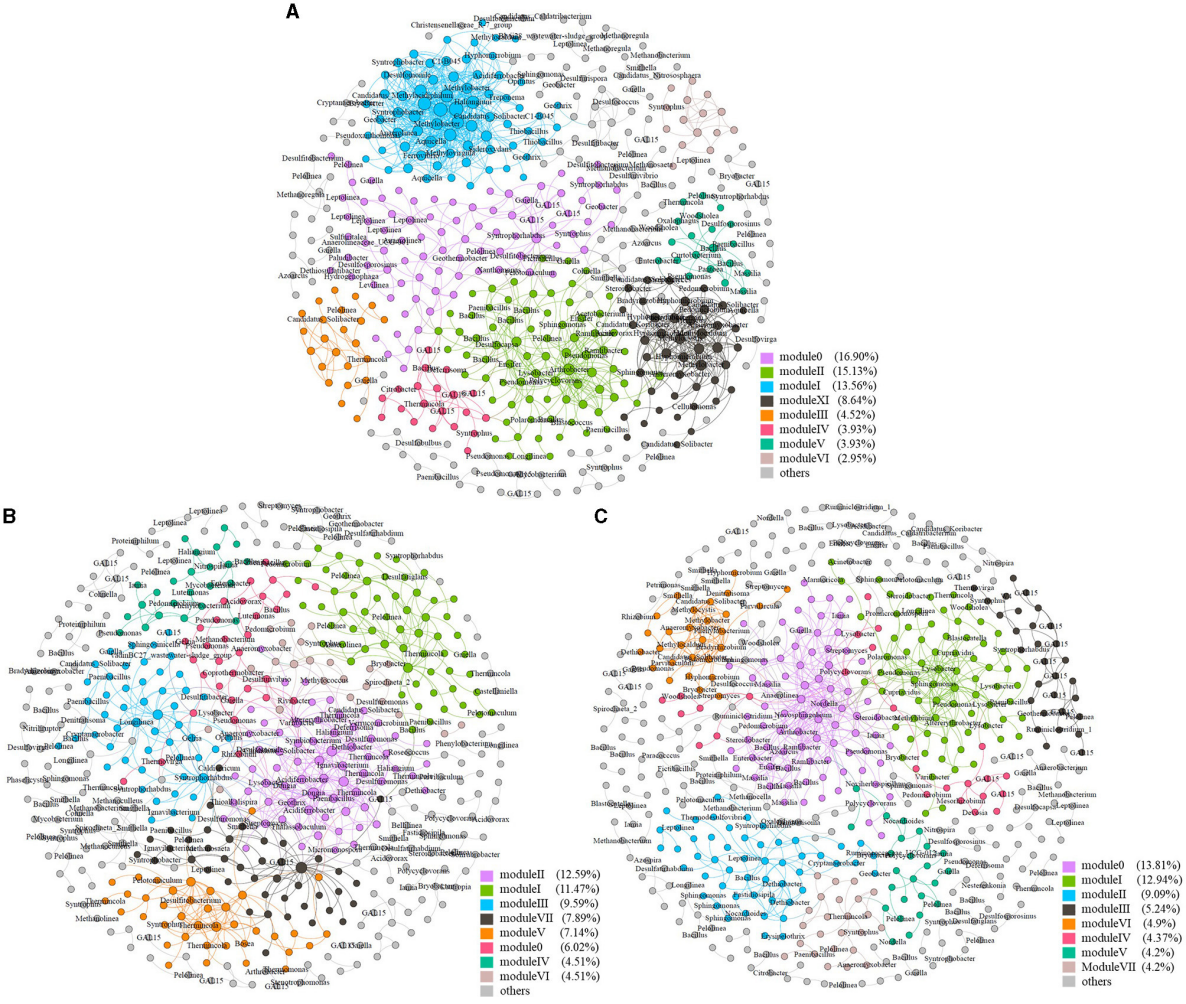
Soil molecular ecological network at different sites. Soil molecular ecological network at site C1 **(A)**, site C2 **(B)**, and site C4 **(C)**.

Three networks were constructed and compared with each other for sampling sites C1, C2, and C4, respectively (Figure 5). The avgK and avgCC, which indicate how nodes are connected and clustered with other nodes (Sheng et al., 2021), were highest at site C1 (Table 4). This suggested that the network at site C1 was more complex and clustered than those at the other sites, although higher pollution levels led to a decrease in microbial α-diversity. Gao *et al*. also found higher avgK and avgCC in heavily contaminated soils compared to uncontaminated soils (Gao et al., 2022). However, different from our results, Geng et al. (2022) found that the avgK and avgCC of both bacterial and fungal communities were lower in heavily contaminated soils (TPH greater than 3,000 mg/kg) than in lightly contaminated soils (TPH greater than 3,000 mg/kg), which suggested that higher pollution levels may lower the microbial network complexity in some conditions.

**Table 4:** Network topological parameters at different sites.

	**Network C1**	**Network C2**	**Network C4**
Samples	20	20	19
Genes	1,092	1,464	1,410
Nodes	509	532	572
Links	1,317	839	905
Cor cutoff	0.78	0.79	0.8
*R*^2^ power-law	0.907	0.93	0.937
avgK	5.175	3.154	3.164
avgCC	0.274	0.193	0.145
GD	6.829	6.551	7.14
HD	5.09	5.247	5.322

All three microbial networks were found to consist of 12–15 modules (more than five nodes), despite the large difference in the number of nodes. The modules in the networks from different sites showed different patterns and relationships with soil organic contaminants. For instance, Module X at site C1, which was composed of species from the Syntrophus genus, Comamonadaceae family, and Lineage_IV order, was found to be positively correlated with soil benzene, toluene, and o-xylene, but negatively correlated with naphthalene (*P* < 0.05) (Supplementary Table S4). Meanwhile, Module IV at site C2 was the only major module that showed a significant correlation with soil C10–C40 (positive correlation, *P* < 0.05) among all the sites (Supplementary Table S5). At site C4, both Module IV and Module V were positively correlated with soil C6–C9, m- and p-xylene, o-xylene, and ethylbenzene, while negatively correlated with toluene (*P* < 0.05) (Supplementary Table S6).

### 3.5. Major network modules and their potential functions

In addition to direct associations between modules and pollutants, some nodes within major modules were found to be involved in the metabolism of different pollutants. The major modules of the network at site C1 included Module I, Module II, and Module XI (Figure 5A). Module I was mainly composed of α-Proteobacteria, δ-Proteobacteria, and γ-Proteobacteria within the Proteobacteria phylum. Among the nodes of α-proteobacteria within Module I, Methylovirgula (Vorob'ev et al., 2009) genus was methylotrophic and Ferrovibrio (Sorokina et al., 2012) genus was a typical neutrophilic Fe(II)-oxidizing bacterium. Within the δ-proteobacteria nodes of Module I, the Haliangium (Fudou et al., 2002) genus was halophilic while Gobacter (Coates et al., 2001) genus could oxidize volatile fatty acids and some aromatic compounds using Fe(III) as electron acceptors. Syntrophobacter (Chen et al., 2005) genus within the δ-proteobacteria nodes was reported to degrade propionate in syntrophic association with methanogens, and Desulfomonile (Alves et al., 2020) genus could degrade long-chain fatty acids using sulfate as electron acceptors. In addition, Methylobacter (Wartiainen et al., 2006) genus within the γ-proteobacteria nodes was a methane-oxidizing bacterium. Module II mainly consisted of β-Proteobacteria and γ-Proteobacteria within the Proteobacteria phylum. Among them, the Ramlibacter (Xie et al., 2010) genus of β-proteobacteria could degrade m-xylene, and Acidovorax (Byrne-Bailey et al., 2010) genus was able to oxidize Fe(II) on nitrate reduction. Polycyclovorans (Gutierrez et al., 2013), a genus of γ-proteobacteria identified within Module II, was a strictly aerobic, halotolerant bacterium that could degrade aliphatic and aromatic hydrocarbon compounds, while Pseudomonas (Barathi and Vasudevan, 2001) and Lysobacter (Maeda et al., 2009) genus within Module II could degrade petroleum hydrocarbons and PAHs, respectively. Different from Modules I and II, more methylotrophic and methanotrophic bacteria were identified within the nodes of Module XI. For example, Hyphomicrobium (Jeong and Kim, 2019) genus was methylotrophic, and methylocystis (Lindner et al., 2007) was methanotrophic. The phenylobacterium (Li et al., 2019) genus identified within Module XI could degrade phenyl compounds. In addition, methylobacter (Wartiainen et al., 2006) and methylocaldum (Takeuchi and Yoshioka, 2021) genera identified within Module XI were both methanotrophic. It is worth noting that methylotrophs could establish cross-feeding associations with methanotrophs in methane oxidation processes. The process in which methanol was finally oxidized to CO_2_ was shared by methylotrophs (Jeong and Kim, 2019). In addition, it was concluded that a module (Module II) specific for the degradation of varying organic pollutants, as well as a module (Module XI) specific for methane and methyl oxidation were formed in the network at site C1.

The network at site C2 comprised several major modules, including Module I, Module II, Module III, and Module V (Figure 5B). Module I was primarily made of Actinobacteria phylum and Anaerolineae class of Chloroflexi phylum. In contrast, Module II consisted mainly of α-Proteobacteria, β-Proteobacteria, δ-Proteobacteria, and γ-Proteobacteria class of the Proteobacteria phylum and Clostridia class of the Firmicutes phylum. Variibacter (Wu et al., 2020) genus was reported to be one of the keystone genera of the microbial network in a brownfield polluted by chlorinated paraffin. Among the nodes of δ-proteobacteria within Module II, Deferrisoma (Perez-Rodriguez et al., 2016) genus was a Fe(III)-reducing bacterium using hydrogen as an energy source. Desulfuromonas (Krumholz, 1997) genus could reduce chlorinated hydrocarbons, while Desulfurivibrio (Poser et al., 2013) genus was a haloalkaliphilic bacterium and involved in the disproportionation of the element sulfur. The Acidiferrobacter (Issotta et al., 2018) genus of γ-proteobacteria was an acidophilic iron and sulfur oxidizer, and Pseudomonas (Barathi and Vasudevan, 2001) genus could degrade petroleum hydrocarbons. Among the nodes of Clostridia within Module II, Thermincola (Toth et al., 2021) genus could degrade benzene under iron-reducing and nitrate-reducing conditions, while Dethiobacter (Sorokin et al., 2008) genus could utilize thiosulfate or polysulfide as electron acceptors. Module III was mainly composed of the Actinobacteria phylum and the Anaerolineae class of Chloroflexi phylum. Longilinea (Yamada et al., 2007) genus of Anaerolineae class was reported to be a member of methanogenic propionate-degrading consortia. Module V mainly consisted of the Actinobacteria phylum and the Clostridia class of Firmicutes phylum. Among nodes of the Clostridia class, Thermincola (Toth et al., 2021) genus could degrade benzene under iron-reducing and nitrate-reducing conditions, and Desulfitobacterium (Futagami et al., 2006) genus exhibited active dechlorinating ability for tetrachloroethene.

The network at site C4 was composed of two major modules: Module 0 and Module I (Figure 5C). Module 0 consisted mainly of α-Proteobacteria and β-Proteobacteria of the Proteobacteria phylum. Within the nodes of α-Proteobacteria, in Module 0, Novosphingobium (Li et al., 2012) genus was capable of degrading certain mono- or polycyclic aromatic hydrocarbons. In addition, the Azoarcus (Junghare et al., 2016) genus of β-Proteobacteria could degrade benzoate or o-phthalate using nitrate as electron acceptors, while Ramlibacter (Xie et al., 2010) and Massilia (Wang et al., 2016) genus could degrade m-xylene and phenanthrene, respectively. In contrast, Module I mainly consisted of β-Proteobacteria, γ-Proteobacteria, and Acidobacteria phylum. Among the nodes of β-Proteobacteria within Module I, Cupriavidus (Perez-Pantoja et al., 2008) genus could degrade some chloroaromatics, while the methylotrophic Methylibium (Song and Cho, 2007) genus was reported to degrade methyl tert-butyl ether, a common gasoline additive. In Module I, the Pseudomonas (Barathi and Vasudevan, 2001) genus of γ-proteobacteria was capable of degrading petroleum hydrocarbons, and Lysobacter (Maeda et al., 2009) genus was able to degrade PAHs. Therefore, Module I might be specific to the degradation of various organic pollutants.

Two parameters, within-module connectivity (*Z*_*i*_) and among-module connectivity (*P*_*i*_) were used to describe the topological roles of the zOTUs (Zhou et al., 2011). In the network at site C1, there were seven nodes as connectors with a high *P* but a low *Z* value, including zOTU_27, zOTU_51, zOTU_54, zOTU_55, zOTU_65, zOTU_119, and zOTU_217. Only zOTU_1 with a low *P* but a high *Z-*value was identified as a module hub in the network at site C1. Among these nodes, zOTU_54 was affiliated with the Arthrobacter (Bae et al., 1996) genus, known for its ability to degrade 4-chlorophenol, while zOTU_217 was affiliated with the bacillus (Varjani, 2017) genus, capable of degrading aliphatic and mono- and polycyclic aromatic hydrocarbons. The only module hub, zOTU_1 was affiliated with the Smithella (Gray et al., 2011) genus, a common alkane-degrading bacterium. In contrast, more connectors and module hubs were found in the network at site C2. There were 10 nodes as connectors, including zOTU_32, zOTU_56, zOTU_123, zOTU_150, zOTU_151, zOTU_167, zOTU_245, zOTU_254, zOTU_492, and zOTU_650, while four module hubs were zOTU_460, zOTU_519, zOTU_1131, and zOTU_1702. Among the connectors of the network at site C2, zOTU_123 was affiliated with the Syntrophorhabdus (Nobu et al., 2014) genus, known for its anaerobic degradation of aromatic compounds like phenol and benzoate in syntrophic associations with some methanogens. zOTU_151 was affiliated with the Longilinea (Yamada et al., 2007) genus, a member of methanogenic propionate-degrading consortia. Furthermore, zOTU_167 was affiliated with the Dongia (Qun et al., 2019) genus, a nitrogen-fixing bacterium in soil, and zOTU_245 was affiliated with the Pseudomonas (Barathi and Vasudevan, 2001) genus, capable of degrading petroleum hydrocarbons. Two of the module hubs, zotu519 and zotu1131, were affiliated with Thermincola (Toth et al., 2021) and Giella (Lu et al., 2019) genera, respectively. The former could degrade benzene under iron-reducing and nitrate-reducing conditions, while the latter could degrade gelatin, which was composed of some alkyl and heterocyclic groups. Seven nodes were also identified as connectors in the network at site C4, including zOTU_84, zOTU_97, zOTU_128, zOTU_160, zOTU_322, zOTU_340, and zOTU_388, while module hubs in the same network included zOTU_419, zOTU_442, and zOTU_844. Among these connectors, zOTU_84 was affiliated with Thermincola (Toth et al., 2021) genus, which could degrade benzene under iron-reducing and nitrate-reducing conditions. zOTU_340 was affiliated with Lysobacter (Maeda et al., 2009) genus, which can degrade PAHs. Among module hubs of the network at site C4, zOTU_419 was affiliated with the denitrifying bacterium Denitratisoma (Cai et al., 2022) genus, while zOTU_442 was affiliated with the hydrogenotrophic methanogen, Methanocella (Sakai et al., 2008) genus. No network hubs with both a high *P-* and high *Z-*values were found in all three networks. Furthermore, no node was found to be a connector or module hub in more than one network, indicating significant differences in topological compositions among networks under different sites and pollution conditions.

Similar modules to Module XI, specific for methane and methyl oxidation in the C1 network of an organic polluted site, have rarely been reported in previous studies. The presence of this module, coupled with the significantly higher relative abundance of methanotrophs and methylotrophs at site C1, indicated stronger methanotrophic and methylotrophic metabolic activities at the heavily polluted site. This could be due to the ease of forming an anaerobic methane-producing metabolic environment under heavier pollution levels. In addition, we found that major modules of the network at site C1 involved more nodes that could utilize varying electron acceptors, such as oxygen, nitrate, iron, and sulfate. The increased network complexity observed under higher pollution levels in our study could be attributed to more metabolic pathways and processes, and more microbial interactions during these metabolic processes. For instance, cross-feeding associations between methylotrophs and methanotrophs may contribute to the increased network complexity at higher pollution levels.

## 4. Conclusion

In this study, we analyzed the shifts in soil microbial diversity, community structure, network complexity, and network ecological function under different levels of petroleum hydrocarbon pollution. Our results suggest that soil microbial α-diversity decreased under high C10–C40 levels, while network complexity increased, indicating more complex microbial potential interactions. We also found a module specific for methane and methyl oxidation under high C10–C40 levels, suggesting stronger methanotrophic and methylotrophic metabolic activities at the heavily polluted site. The increased network complexity observed may be attributed to more metabolic pathways and processes, as well as more microbial interactions during these processes. These findings highlight the importance of considering both microbial diversity and network complexity in assessing the impact of pollution on soil ecosystems.

## Data availability statement

The datasets presented in this study can be found in online repositories. The names of the repository/repositories and accession number(s) can be found below: https://www.ncbi.nlm.nih.gov/, no. PRJNA947495.

## Author contributions

Research questions and experimental strategy were developed by YY, XG, and GL. Sample collection, DNA preparation, and sequencing analysis were carried out by JZhu, RZ, YZ, and TD. Soil chemical analyses were carried out by JZhu. Various statistical analyses were carried out by JZhu, RZ, YZ, TD, ZY, and QG. Assistance in data interpretation was provided by YY, XG, GL, and JZho. The manuscript was written by JZhu with the help of YY, XG, and GL. All authors contributed intellectual input and assistance to this study.
