# Supplementary material for: Petroleum pollution changes microbial diversity and network complexity of soil profile in an oil refinery
Source: Front Microbiol. 2023 May 23;14:1193189. doi: 10.3389/fmicb.2023.1193189 (PMC10242060; doi:10.3389/fmicb.2023.1193189)
Supplement: Supplementary file 1 [file Table_1.DOCX]

**Table S1. Distribution of soil samples in each borehole.** Samples failing in DNA extraction are underlined.

| borehole | depth/m | sample name |
| --- | --- | --- |
| C1D0 | 0.5, 1.5, 3, 4, 4.5 | C1D0_0.5, C1D0_1.5, C1D0_3, C1D0_4, C1D0_4.5 |
| C1D1 | 0.5, 1.5, 3, 4, 5 | C1D1_0.5, C1D1_1.5, C1D1_3, C1D1_4, C1D1_5 |
| C1D2 | 0.5, 1.5, 2.5, 4, 5 | C1D2_0.5, C1D2_1.5, C1D2_2.5, C1D2_4, C1D2_5 |
| C1D3 | 0.5, 1.5, 3, 4, 4.5 | C1D3_0.5, C1D3_1.5, C1D3_3, C1D3_4, C1D3_4.5 |
| C2D0 | 0.5, 1.5, 2.5, 3.5, 4.5 | C2D0_0.5, C2D0_1.5, C2D0_2.5, C2D0_3.5, C2D0_4.5 |
| C2D1 | 0.5, 1.5, 3, 4, 4.5 | C2D1_0.5, C2D1_1.5, C2D1_3, C2D1_4, C2D1_4.5 |
| C2D2 | 0.5, 1.5, 3, 3.5, 4.5 | C2D2_0.5, C2D2_1.5, C2D2_3, C2D2_3.5, C2D2_4.5 |
| C2D3 | 0.5, 2, 3, 4, 4.5 | C2D3_0.5, C2D3_2, C2D3_3, C2D3_4, C2D3_4.5 |
| C3D1 | 0.5, 2, 2.5, 4, 4.5 | C3D1_0.5, C3D1_2, C3D1_2.5, C3D1_4, C3D1_4.5 |
| C3D2 | 0.5, 1.5, 3, 3.5, 4.5 | C3D2_0.5, C3D2_1.5, C3D2_3, C3D2_3.5, C3D2_4.5 |
| C3D3 | 0.5, 2, 2.5, 4, 5 | C3D3_0.5, C3D3_2, C3D3_2.5, C3D3_4, C3D3_5 |
| C4D0 | 0.5, 2, 3, 4, 5 | C4D0_0.5, C4D0_2, C4D0_3, C4D0_4, C4D0_5 |
| C4D1 | 0.5, 2, 2.5, 3.5, 4.5 | C4D1_0.5, C4D1_2, C4D1_2.5, C4D1_3.5, C4D1_4.5, |
| C4D2 | 0.5, 2, 3, 4, 5 | C4D2_0.5, C4D2_2, C4D2_3, C4D2_4, C4D2_5 |
| C4D3 | 0.5, 2, 2.5, 3.5, 5 | C4D3_0.5, C4D3_2, C4D3_2.5, C4D3_3.5, C4D3_5 |

**Table S2. Pearson's correlation analysis (R value) between organic contaminants and soil environmental variables.** Significances are denoted by bold type.

|  | C10-C40 | C6-C9 | benzene | toluene | ethylbenzene | m-&p-xylene | o-xylene | naphthalene |
| --- | --- | --- | --- | --- | --- | --- | --- | --- |
| pH | -0.193 | -0.016 | -0.021 | -0.044 | -0.046 | -0.029 | -0.043 | -0.070 |
| depth | -0.010 | **0.240** | **0.243** | **0.248** | **0.256** | **0.268** | **0.278** | 0.078 |
| moisture | -0.082 | -0.220 | 0.062 | -0.063 | -0.188 | -0.209 | -0.183 | -0.228 |
| clay | 0.170 | -0.135 | **0.436** | -0.011 | -0.068 | -0.077 | -0.031 | -0.036 |
| silt | 0.097 | -0.138 | 0.222 | 0.019 | -0.023 | -0.073 | -0.009 | **-0.249** |
| sand | -0.125 | 0.152 | **-0.298** | -0.014 | 0.036 | 0.082 | 0.016 | 0.223 |
| conductivity | -0.189 | -0.210 | -0.111 | -0.129 | -0.224 | -0.208 | -0.200 | -0.148 |
| SOC | **0.666** | **0.642** | **0.738** | **0.796** | **0.713** | **0.703** | **0.798** | **0.268** |
| TN | **0.423** | -0.148 | -0.032 | -0.060 | -0.155 | -0.151 | -0.117 | -0.052 |
| available N | 0.078 | 0.010 | 0.073 | 0.019 | 0.067 | -0.020 | 0.020 | -0.036 |
| NO_3_^-^-N | **0.237** | -0.002 | 0.080 | 0.019 | 0.039 | 0.027 | 0.032 | 0.233 |
| NH_4_^+^-N | -0.078 | -0.075 | -0.007 | -0.035 | -0.079 | -0.073 | -0.064 | -0.061 |
| TP | **-0.400** | -0.076 | -0.032 | -0.081 | -0.090 | -0.059 | -0.087 | -0.077 |
| available P | -0.102 | -0.103 | -0.053 | -0.046 | -0.094 | -0.097 | -0.083 | -0.068 |
| F | -0.008 | **-0.353** | -0.226 | **-0.287** | **-0.368** | **-0.353** | **-0.374** | -0.043 |
| CEC | -0.145 | **-0.247** | -0.061 | -0.141 | **-0.243** | **-0.240** | -0.224 | 0.013 |
| Al | **0.259** | **-0.264** | -0.038 | -0.155 | **-0.244** | **-0.243** | -0.227 | -0.004 |
| Fe | -0.218 | **-0.254** | -0.047 | -0.174 | **-0.250** | **-0.249** | **-0.246** | 0.051 |
| Mn | -0.209 | -0.181 | 0.047 | -0.086 | -0.166 | -0.167 | -0.156 | 0.023 |

**Table S3. Pairwise adonis among different sites based on Bray-Curtis distance**

|  | R square | p value |
| --- | --- | --- |
| C1 vs C2 | 0.129 | 0.001 |
| C1 vs C3 | 0.262 | 0.001 |
| C1 vs C4 | 0.106 | 0.001 |
| C2 vs C3 | 0.207 | 0.001 |
| C2 vs C4 | 0.076 | 0.001 |
| C3 vs C4 | 0.173 | 0.001 |

**Table S4. Pearson's correlation analysis (R value) between network modules and organic contaminants at site C1.** Significances are denoted by bold type.

|  | C10-C40 | C6-C9 | benzene | toluene | ethylbenzene | m-&p-xylene | o-xylene | naphthalene |
| --- | --- | --- | --- | --- | --- | --- | --- | --- |
| module0 | -0.200 | -0.180 | -0.270 | -0.064 | -0.180 | -0.200 | -0.180 | 0.230 |
| moduleⅠ | -0.280 | -0.350 | **-0.560** | **-0.440** | -0.310 | -0.380 | -0.320 | -0.400 |
| moduleⅡ | 0.210 | -0.095 | -0.160 | -0.340 | -0.015 | -0.038 | -0.002 | -0.250 |
| moduleⅢ | -0.430 | -0.170 | -0.027 | 0.120 | 0.015 | -0.041 | 0.140 | **-0.810** |
| moduleⅣ | -0.300 | -0.150 | -0.160 | -0.160 | -0.120 | -0.150 | -0.062 | -0.380 |
| moduleⅤ | -0.130 | 0.270 | 0.300 | 0.340 | 0.250 | 0.260 | 0.280 | -0.230 |
| moduleⅥ | -0.230 | -0.110 | **-0.500** | -0.350 | -0.100 | -0.100 | -0.150 | -0.062 |
| moduleⅦ | 0.013 | -0.080 | -0.420 | -0.270 | -0.100 | -0.140 | -0.120 | -0.160 |
| moduleⅧ | -0.240 | -0.140 | **0.530** | **0.670** | 0.370 | 0.400 | 0.340 | -0.065 |
| moduleⅨ | -0.350 | -0.240 | -0.065 | 0.037 | -0.031 | -0.067 | -0.018 | -0.400 |
| moduleⅩ | 0.320 | 0.390 | **0.570** | **0.460** | 0.430 | 0.420 | **0.520** | **-0.520** |
| moduleⅩⅠ | -0.230 | -0.170 | -0.270 | 0.210 | -0.200 | -0.200 | -0.260 | -0.290 |
| moduleⅩⅤ | 0.024 | 0.130 | 0.230 | 0.390 | 0.180 | 0.180 | 0.220 | -0.410 |

**Table S5. Pearson's correlation analysis (R value) between network modules and organic contaminants at site C2.** Significances are denoted by bold type.

|  | C10-C40 | C6-C9 | benzene | toluene | ethylbenzene | m-&p-xylene | o-xylene | naphthalene |
| --- | --- | --- | --- | --- | --- | --- | --- | --- |
| module0 | 0.007 | 0.430 | 0.054 | 0.380 | 0.150 | 0.360 | **0.740** | 0.410 |
| moduleⅠ | -0.089 | -0.130 | 0.047 | -0.097 | -0.130 | -0.120 | -0.210 | -0.420 |
| moduleⅡ | -0.120 | -0.170 | 0.058 | -0.230 | -0.120 | -0.190 | -0.250 | **-0.780** |
| moduleⅢ | 0.170 | -0.330 | -0.150 | -0.250 | -0.250 | -0.300 | **-0.440** | -0.038 |
| moduleⅣ | **0.710** | -0.060 | -0.110 | -0.190 | -0.039 | -0.090 | -0.220 | 0.370 |
| moduleⅤ | 0.130 | -0.300 | 0.077 | -0.330 | -0.220 | -0.330 | **-0.450** | -0.056 |
| moduleⅥ | 0.008 | -0.130 | **0.550** | -0.190 | -0.100 | -0.130 | -0.250 | -0.380 |
| moduleⅦ | -0.180 | -0.320 | 0.300 | -0.320 | -0.240 | -0.350 | **-0.510** | **-0.720** |
| moduleⅧ | -0.017 | 0.130 | **0.500** | 0.150 | 0.100 | 0.160 | 0.120 | -0.210 |
| moduleⅨ | 0.110 | 0.280 | 0.320 | -0.250 | 0.340 | 0.270 | 0.170 | -0.089 |
| moduleⅩ | 0.170 | 0.260 | 0.250 | -0.280 | 0.410 | 0.310 | 0.150 | -0.082 |
| moduleⅩⅠ | -0.250 | -0.270 | 0.400 | -0.170 | -0.150 | -0.230 | -0.250 | **-0.510** |
| moduleⅩⅡ | -0.250 | -0.310 | 0.230 | -0.190 | -0.260 | -0.300 | -0.240 | -0.340 |
| moduleⅩⅣ | -0.034 | -0.240 | 0.120 | -0.330 | -0.100 | -0.220 | -0.420 | -0.280 |
| moduleⅩⅤ | -0.088 | -0.062 | **0.530** | -0.320 | 0.150 | -0.120 | -0.058 | -0.220 |

**Table S6. Pearson's correlation analysis (R value) between network modules and organic contaminants at site C4.** Significances are denoted by bold type.

|  | C10-C40 | C6-C9 | benzene | toluene | ethylbenzene | m-&p-xylene | o-xylene | naphthalene |
| --- | --- | --- | --- | --- | --- | --- | --- | --- |
| module0 | -0.330 | -0.220 | 0.099 | 0.390 | 0.240 | 0.170 | 0.076 | 0.130 |
| moduleⅠ | -0.048 | -0.240 | -0.410 | -0.039 | **0.470** | -0.072 | 0.290 | **0.520** |
| moduleⅡ | -0.340 | -0.120 | -0.360 | **-0.930** | 0.440 | -0.064 | 0.035 | -0.300 |
| moduleⅢ | -0.340 | -0.240 | **-0.500** | **-0.920** | -0.150 | -0.220 | -0.160 | **-0.630** |
| moduleⅣ | 0.190 | **0.540** | **-0.590** | **-0.960** | **0.600** | **0.740** | **0.660** | 0.095 |
| moduleⅤ | -0.210 | **0.700** | -0.280 | **-0.960** | **0.540** | **0.590** | **0.490** | -0.240 |
| moduleⅥ | 0.042 | **-0.550** | **-0.830** | **-0.810** | **-0.460** | -0.310 | -0.230 | -0.390 |
| moduleⅦ | -0.230 | 0.190 | 0.055 | **-0.970** | -0.035 | -0.033 | 0.300 | -0.390 |
| moduleⅧ | 0.090 | 0.230 | -0.023 | **-0.460** | -0.270 | -0.110 | -0.260 | **-0.470** |
| moduleⅨ | -0.079 | 0.290 | 0.047 | **-0.960** | -0.220 | -0.190 | -0.330 | **-0.490** |
| moduleⅩ | -0.220 | 0.200 | -0.340 | **-0.580** | 0.440 | 0.220 | 0.069 | -0.160 |
| moduleⅩⅠ | 0.220 | **0.690** | 0.330 | **-0.880** | 0.370 | 0.240 | 0.042 | 0.044 |
| moduleⅩⅡ | 0.037 | 0.370 | -0.120 | -0.350 | 0.390 | 0.380 | **0.570** | -0.035 |
